# Translational landscape of glioblastoma immunotherapy for physicians: guiding clinical practice with basic scientific evidence

**DOI:** 10.1186/s13045-022-01298-0

**Published:** 2022-06-11

**Authors:** Daniel Kreatsoulas, Chelsea Bolyard, Bill X. Wu, Hakan Cam, Pierre Giglio, Zihai Li

**Affiliations:** 1 Department of Neurological Surgery, The Ohio State University Wexner Medical Center, Columbus, OH USA; 2 Pelotonia Institute for Immuno-Oncology, The Ohio State University Comprehensive Cancer Center – Arthur G. James Cancer Hospital and Richard J. Solove Research Institute, The Ohio State University, 460 W 12th Avenue, BRT 550, Columbus, OH 43210 USA; 3 Department of Neuro-Oncology, The Ohio State University Wexner Medical Center, James Cancer Center, Columbus, OH USA; 4 Department of Medical Oncology, The Ohio State University Wexner Medical Center, James Cancer Center, Columbus, OH USA

**Keywords:** Glioblastoma, Immunotherapy, Tumor microenvironment, Immuno-oncology, Immune checkpoint inhibitors, Dendritic cell vaccines, Peptide vaccines, CAR-T cells

## Abstract

Despite recent advances in cancer therapeutics, glioblastoma (GBM) remains one of the most difficult cancers to treat in both the primary and recurrent settings. GBM presents a unique therapeutic challenge given the immune-privileged environment of the brain and the aggressive nature of the disease. Furthermore, it can change phenotypes throughout the course of disease—switching between mesenchymal, neural, and classic gene signatures, each with specific markers and mechanisms of resistance. Recent advancements in the field of immunotherapy—which utilizes strategies to reenergize or alter the immune system to target cancer—have shown striking results in patients with many types of malignancy. Immune checkpoint inhibitors, adoptive cellular therapy, cellular and peptide vaccines, and other technologies provide clinicians with a vast array of tools to design highly individualized treatment and potential for combination strategies. There are currently over 80 active clinical trials evaluating immunotherapies for GBM, often in combination with standard secondary treatment options including re-resection and anti-angiogenic agents, such as bevacizumab. This review will provide a clinically focused overview of the immune environment present in GBM, which is frequently immunosuppressive and characterized by M2 macrophages, T cell exhaustion, enhanced transforming growth factor-β signaling, and others. We will also outline existing immunotherapeutic strategies, with a special focus on immune checkpoint inhibitors, chimeric antigen receptor therapy, and dendritic cell vaccines. Finally, we will summarize key discoveries in the field and discuss currently active clinical trials, including combination strategies, burgeoning technology like nucleic acid and nanoparticle therapy, and novel anticancer vaccines. This review aims to provide the most updated summary of the field of immunotherapy for GBM and offer both historical perspective and future directions to help inform clinical practice.

## Introduction

Gliomas are primary cancers of the central nervous system (CNS) and are comprised of several distinct subtypes. They are the most common brain tumors in adults (6 per 100,000 people for all gliomas), and the most common subtype glioblastoma (GBM) is also the most lethal (incidence, 3 per 100,000 people) [1]. Gliomas can be organized into three main progenitor cell classes: astrocytomas, ependymomas, and oligodendrogliomas. These subtypes are genetically and clinically distinct and respond differently to treatment strategies. Generally, ependymoma is considered separately from astrocytoma and oligodendroglioma. Astrocytomas are the more common variety of the two (1.3 per 100,000 (astrocytoma) vs 0.5 per 100,000 (oligodendroglioma)) [1]. Gliomas have historically had a very poor prognosis, with the most aggressive subtype, GBM having a dismal median survival time of approximately 1 year. While outcomes have improved in the last few decades [2, 3], by a few months, current treatment strategies leave much to be desired.

The molecular biology and cellular identity of gliomas, particularly GBM, have been highly studied over the last few decades, with promising advancements [4, 5]. The World Health Organization (WHO) has revised its classification of gliomas in 2016 [6], which took into account the improved understanding resulting from advances in genomic medicine that occurred over the previous 20 years. The major subclassifications combine both gross pathologic and molecular diagnostic criteria, as summarized by various excellent reviews [4–6]. In general, grade II and III astrocytomas are 1p/19q non-deleted and either IDH-1 (isocitrate dehydrogenase-1) wild type or mutant, whereas grade II and III oligodendrogliomas are 1p/19q co-deleted and IDH-1 mutant [6, 7]. GBM was classified as either *primary* (arises de novo, IDH wild type, with mutations in telomerase reverse transcriptase [TERT], epidermal growth factor receptor [EGFR], and phosphatase and tensin homolog [PTEN]), or *secondary* (progresses from a grade II or III astrocytoma, IDH mutant, with mutations in tumor protein 53 [TP53], and alpha-thalassemia/mental retardation syndrome X-linked [ATRX] gene). Recent updates focus on differences in biology that correlate with IDH status to delineate IDH mutant tumors (astrocytoma WHO Grade IV) vs IDH wild-type tumors (glioblastoma) [8, 9].

GBM is the most commonly diagnosed intracranial glioma, accounting for about half of all new diagnoses in the USA each year [6, 7]. This is also the most aggressive type of glioma, with 5-year survival rates ranging from 0.5 to 5.8%, depending on the study [2, 7]. Some prognostic factors correlate with improved survival, including a higher Karnofsky performance status (KPS) score (> 70 is beneficial), extent of initial tumor resection (gross total resection is the standard of care), and younger age at diagnosis (< 65 years) [10–12]. Unfortunately, despite an improved understanding of etiology and outcomes, GBM has a population median survival of only 13.5 months with aggressive therapy [2].

### Standard-of-care treatment

Standard-of-care treatment for newly diagnosed GBM includes maximal safe surgical resection followed by fractionated radiotherapy of 60 Gy in 30 fractions with concurrent temozolomide (TMZ) chemotherapy, a DNA-methylating agent, and tumor-treating fields [2, 13]. Tumor resection is based on imaging characteristics, and a standard “gross total” resection refers to removal of the contrast-enhancing and necrotic central portions of the tumor on MRI (magnetic resonance imaging) or CT (computed tomography) scan [14, 15]. Despite aggressive resection techniques, GBM will almost always recur due to tumor infiltration along normal white matter tracts in the brain, which can be visualized via MRI as edematous regions in adjacent or distant parenchyma. This T2 intense/FLAIR (fluid-attenuated inversion recovery) hyperintense of a scan region contains viable tumor cells, which are treated using postoperative chemotherapy and radiation protocols, even in gross total resections. Unfortunately, supratotal resection that includes part of the FLAIR hyperintense region has not been shown to improve survival beyond that of a traditional gross total resection [14].

Other strategies that improve survival in some patients are considered extended standard-of-care protocols. For example, the anti-vascular endothelial growth factor (VEGF) antibody bevacizumab (Avastin®) has shown improved progression-free survival and overall survival in clinical trials when used in GBM. This treatment is approved for use by the Food and Drug Administration (FDA) in recurrent GBM and is often used off-label for treatment of radiation necrosis [16, 17]. A second adjunct technology, tumor-treating fields, administers electromagnetic pulses that interfere with mitotic bundling in the tumor through a device worn on the head. This treatment has shown some survival benefit and is now FDA-approved and included in the standard of care [18, 19]. However, with maximal standard treatment including tumor-treating fields, the median survival has only extended to a maximum of 20.7 months in clinical trials based on a recent systematic review [2, 16, 20–22].

### The unique immunosuppressive talents of GBM

Given the limited impact of standard-of-care therapy on survival, immunotherapy and genetic/personalized medicine may be the future of GBM treatment. Unfortunately, due to low mutational burden on average and the isolation of the central nervous system (CNS), GBM does not present a significant amount of neoantigen to immune surveillance mechanisms, making it difficult to target in a broad immunological sense, such as with checkpoint inhibition [23, 24]. In comparison with other tumor types, GBM as a whole has a low degree of mutational or deletional neoantigen, generally in the bottom half of broad tumor categories [25]. However, even when mutational burden is moderate or high, there is not a meaningful increase in checkpoint molecule expression or immune cell infiltration [24, 25]. Genetic mutation, either intrinsic or acquired following the use of alkylating agents (such as TMZ), can result in a more immunosuppressive tumor microenvironment (TME) [23, 26]. Additionally, since GBM can be heterogeneous, individual tumors may contain immunologically distinct subtypes, each of which will respond differently to treatment [23, 26].

Generally, GBM can be classified into three genetic profiles: proneural/neural, classical, and mesenchymal, correlating to changes in the gene expression patterns of platelet-derived growth factor receptor alpha (PDGFRA)/IDH1, EGFR, and neurofibromatosis type 1 (NF1), respectively [27, 28]. Each subtype has distinct genetic and phenotypic signatures in the immune microenvironment that alter their behavior [29]. The proneural/neural and classical subtypes tend to have a lesser degree of immunosuppression and also fewer proinflammatory genes activated vs the mesenchymal subtype, and they may exist in the tumor alongside the mesenchymal subtype [27]. Of the subtypes, mesenchymal is considered the most aggressive and can be induced by DNA damage or the infiltration and signaling cascades of innate immune cells, particularly immunosuppressive M2 macrophages [26, 30, 31]. Mesenchymal transition from another subtype is associated with highly elevated levels of tumor-associated macrophages (TAMs) and tumor-infiltrating T cells [31]; the upregulation of the NF-κB pathway via increased TNF-α (tumor necrosis factor-α) signaling in the TME; and deactivation of NF1 [26, 32]. The immunological differences between subtypes are underlined by the significant variations in response to treatment [27]. Moreover, analysis of the immune environment transcriptome has determined that differences in GBM can occur in short-term vs long-term relapse, regardless of therapy such as radiation and temozolomide-based chemotherapy [26]. GBM is a very adaptive tumor that quickly responds to treatment pressure via genotypic and phenotypic changes, which contributes to its high recurrence rates.

The CNS offers some advantages to GBM because it is an immunologically isolated and relatively privileged location, according to traditional doctrine. However, recent evidence has shown that there is a connection between the CNS and the systemic immune environments, and studies indicate that robust immune responses do exist in the brain. The classic Medawar experiment showed that a skin allograft placed in the brain does not trigger significant systemic immune response unless there was a prior injection of a peripheral allograft [33]. This suggests that the systemic immune system can be primed to respond to antigens located in the CNS under specific conditions. While antigen presentation is limited in the brain, there is an active connection between the brain tissue and the lymphatic system through glymphatic channels, which were discovered via MRI in 2018 [34]. Additionally, meningeal drainage to the systemic lymph via the cervical lymph nodes allows for immunological surveillance of the brain tissue [35–38]. These glymphatic channels likely house the dendritic cell and neutrophil nests that are implicated in autoimmune neurologic inflammation and antigen presentation to the peripheral immune system; these nests are potential targets for future novel therapeutics [39, 40]. Neutrophils have been preliminarily implicated in helping drive GBM progression through ferroptosis mechanisms [41]. The role that dendritic cells and neutrophils play in GBM progression and the TME remains poorly understood and an area of active investigation, as summarized by excellent reviews [42, 43].

Moreover, the glioma cells induce changes in the local environment that result in chemoresistance and immune escape, both via signaling and cytoarchitecture alterations. For instance, tumor cell proliferation can create new basement membrane, extracellular matrix components, and new blood vessels de novo, secondary to the downstream effects of tumor-associated fibroblastic cells and the secretion of VEGF and other factors. This re-engineering protects the tumor against treatment and immune invasion. For example, pericytes are a key component of the blood–brain barrier (BBB) and often derive from GSCs (glioma stem cells) [44]. Additionally, it has been shown that while some portions of each GBM have a disrupted BBB (for example, surrounding the highly necrotic areas), there are portions with an intact barrier, creating nests of cells that are highly resistant to many chemotherapeutic drugs [45]. Together, these and other factors lead to incredible resistance to conventional chemotherapy among glioma. Significant efforts to induce leakiness of the BBB via various mechanisms and induce therapeutic sensitivity utilize various mechanisms including targeting pericytes [46], ultrasound treatments [47], and nanoparticles [48], and some ongoing clinical trials in glioma involve these approaches [NCT02766699].

**Fig. 1:**
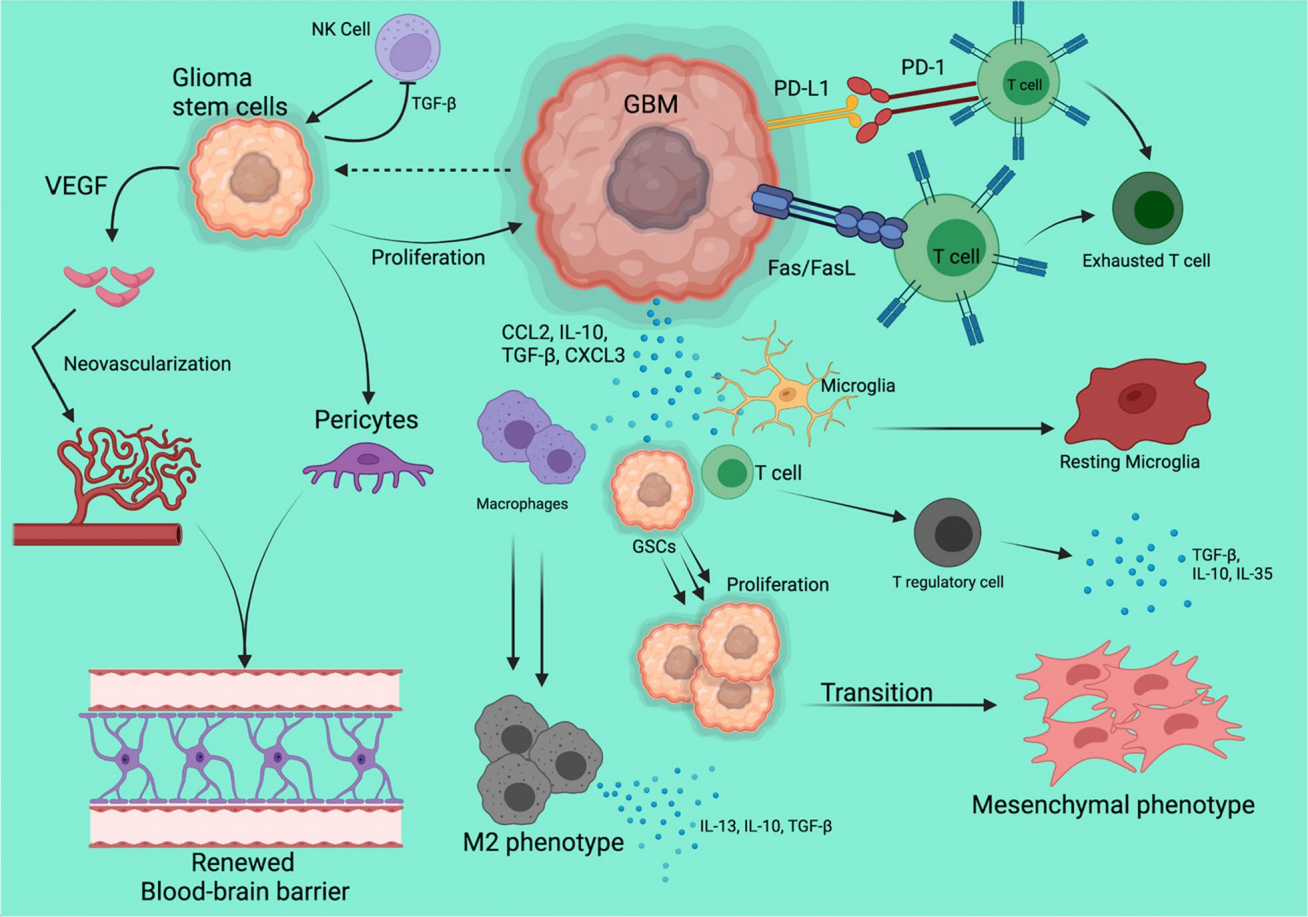
Visual representation of the immune escape mechanisms of glioblastoma. Utilizing a combination of glioma stem cell actions, cytokine and molecular signaling, and direct cell–cell interactions, the tumor cells are able to evade immune cells and continue their proliferation. Figure made and published via paid subscription to Biorender.com

In addition to inducing structural changes, tumor cells can alter the immune compartment of the TME and create an immunosuppressive environment that promotes therapeutic resistance, tumor aggression, and tumor cell mobility and proliferation. For example, a large proportion of gliomas secrete IL-33 (interleukin-33), which promotes recruitment of innate immune cells to create a tumor-promoting microenvironment [49]. Location within the relatively immune-privileged CNS is key to GBM resistance to immunotherapy, and significant work is ongoing to study the components of the environments mediating this resistance. The mechanisms employed by GBM to induce an immunosuppressive environment and reduce immune surveillance are quite plentiful and are summarized in Fig. 1 and described below.

One specific pathway that has been significantly explored in GBM is the TGF-β (transforming growth factor-β) signaling pathway. It is known to be an active protumorigenic cytokine within the brain TME [50–52]. There is evidence that GBM can acquire mutations that allow escape from TGF-β tumor suppressive effects and actively encourage the protumorigenic effects [50, 53–55]. TGF-β signaling is important in invasiveness, angiogenesis, and local immune suppression for GBM tumor cells. In addition, it works through a variety of mechanisms to maintain the stemness of GSCs [50, 51, 53].

TGF-β comes in three isoforms, TGF-β1/2/3, where each has distinctive roles within the signaling pathways for different cells and cell types [55]. The molecule’s signaling pathway is dependent on a membrane serine/threonine kinase complex that phosphorylates members of the Smad family of proteins to drive transcriptional changes in the cell [55–57]. There are a variety of TGF-β receptors and Smad proteins, allowing TGF-β to have a variety of effects within the body [55, 58]. TGF-β increases GBM tumor invasiveness through deregulation of growth factor receptor expression, increasing levels of VEGF secreted by the tumor, and differentiating local epithelial cells into mesenchymal-type cells with reduced expression of cell adhesion molecules [51, 53, 59].

Pools of TGF-β exist in the cellular milieu in latent forms, namely the large latent complex tied to the extracellular matrix [55, 60], or tied to the glycoprotein-A repetitions predominant (GARP) transmembrane domain, expressed on effector immune cells such as Tregs, B cells, and platelets [61–65]. GARP acts as a membrane-bound and soluble activator of TGF-β via dissociation of latency-associated peptide (LAP) from TGF-β molecules, enabling signaling through receptor binding [65]. GARP acts as a potentiator of active TGF-β release from regulatory T cells in the human body and normally promotes immune tolerance [66, 67]. However, it has been shown to be significantly upregulated in GBM and other aggressive human cancers, and research has linked it to promoting an epithelial-to-mesenchymal transition and increased tumor immune evasion [62, 65, 67–70]. Along the same vein, platelets have been extensively shown to suppress CD8 + T cell immunity in the context of TGF-β signaling via GARP and actively promote cancer progression and aggressiveness, but can be targeted in vivo to enhance tumor control [61, 65, 71]. There has been evidence published as well that GBM patients have higher peripheral platelet counts, especially when the tumor is progressing [72, 73]. These findings may explain some facets of GBM immune evasion because of the significant degree of intratumoral hemorrhage and necrosis, spilling platelets into the tumor microenvironment.

TGF-β and its downstream signaling pathway have significant immunosuppressive effects on both the adaptive and innate immune systems, especially in the GBM TME. Among the various mechanisms that GBM can manipulate, TGF-β pathways are among the best studied, but active ongoing research continues to uncover other mechanisms of promise, discussed later in the review.

Because of the relative lack of efficacy of prior therapies, there have been many attempts at novel treatments, looking for therapeutic potential in immunotherapy, vaccines, and other modalities. We will review the major categories of these potential therapies and the science behind them.

### Immunotherapy for GBM

#### Immune checkpoint inhibitors

**Fig. 2:**
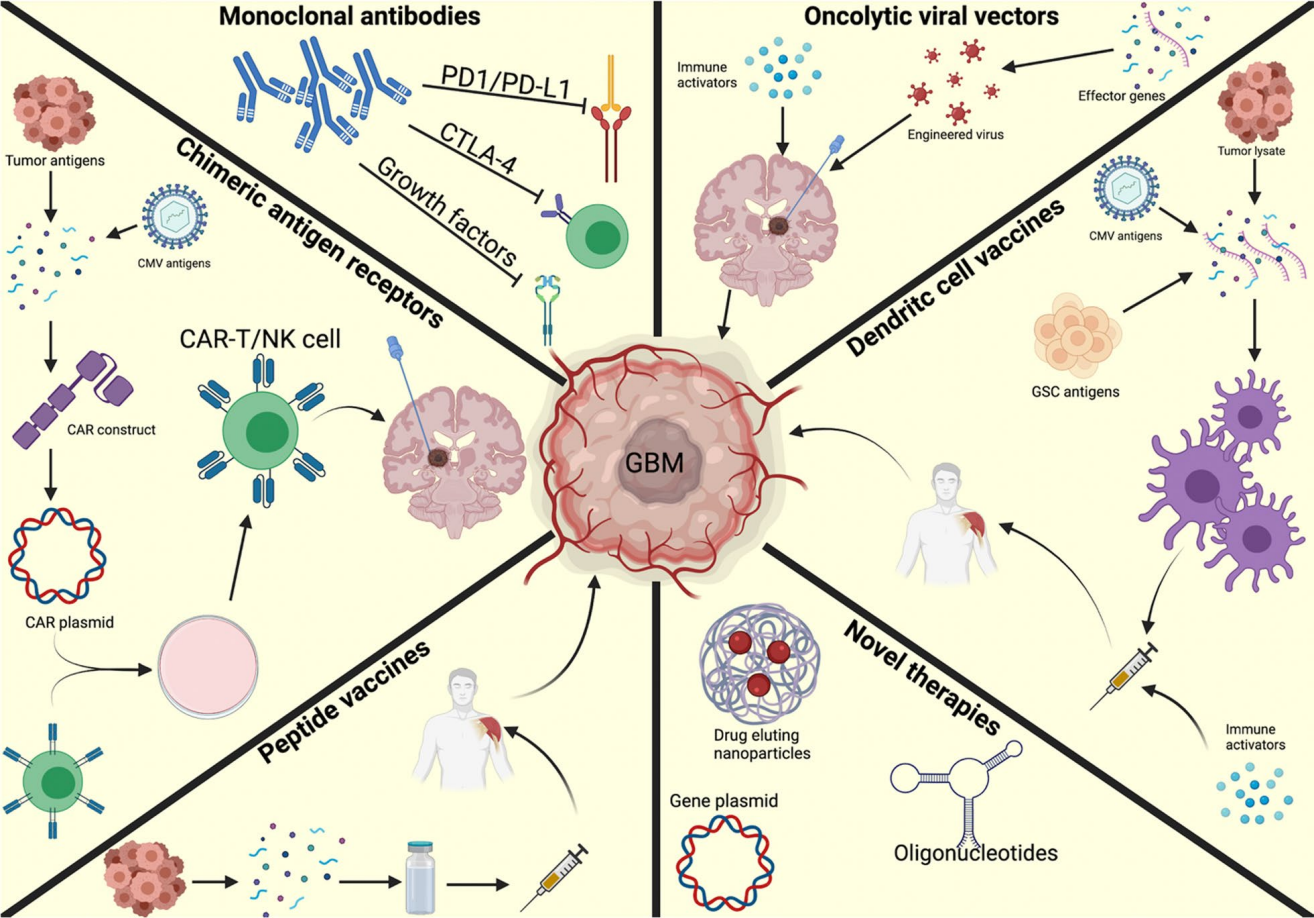
Visual representation of various available immunotherapies that have been studied in glioblastoma. Figure made and published via paid subscription to Biorender.com

Immune checkpoint inhibitors (ICIs) represent the most widely studied category of immunotherapeutics for GBM (Fig. 2). ICIs have shown efficacy in clinical trials for many different malignancies, both in adjuvant and in neoadjuvant settings, with overall durable results [74–79]. Profound responses have been seen in patients with melanoma and non-small cell lung carcinoma (NSCLC) using therapies targeting the programmed death (ligand)-1 (PD[L]-1) and cytotoxic T-lymphocyte antigen-4 (CTLA-4) signaling pathways [77]. PD-1 is a surface receptor expressed by activated T cells that interacts with PD-L1, which is expressed by a wide range of cells within the body [80]. The binding of PD-1/PD-L1 down-regulates the T cell receptor (TCR) and CD28 signaling (among other mechanisms), which inhibits T cell effector activities and drives the development of an immunosuppressive environment [80–83]. GBM expresses elevated levels of PD-L1, making it an attractive potential target for immunotherapy trials [84–86]. And indeed, anti-PD-1 treatment has been shown in a laboratory setting to allow transition from an M2 to an M1 macrophage phenotype and a proinflammatory environment, providing promising translational background [87]. However, another group demonstrated that while CD8 + T cell and aDC1 activation improved with anti-PD-1 treatment, the immunotherapy was not able to overcome this M2 macrophage polarization [88]. In spite of promising background science, PD-1/PD-L1 immunotherapy trials with GBM have not been fruitful to date.

The first extensive evaluation of PD-1/PD-L1 immunotherapy in GBM was the CheckMate 143 trial, which evaluated 369 patients with recurrent GBM who received either nivolumab (an anti-PD-1 monoclonal antibody) or bevacizumab [17]. There was no significant difference in median overall survival (OS) (9.8 months nivolumab vs 10.0 months bevacizumab), and the objective response rate to treatment was higher in the bevacizumab group. Nearly 1 in 5 patients in this trial had treatment-related adverse events [17]. Two ongoing trials are currently evaluating the role of nivolumab in newly diagnosed GBM: CheckMate 548, in which patients receive either standard of care (RT + TMZ) or standard of care plus nivolumab; and CheckMate 498, in which patients receive either RT + TMZ or RT + nivolumab [81]. As of 2020, however, Bristol Myers-Squibb announced that the CheckMate 548 trial would not meet its primary endpoint (progression-free survival [PFS] and OS) based on an independent review of their data in the overall randomized population [89]. While many trials are still underway to evaluate the impact of checkpoint inhibitors on GBM, it was discovered that TMZ therapy can disrupt PD-L1 expression in recurrent GBM [90, 91], which may explain the negative results seen in trials evaluating nivolumab for recurrent GBM. Analysis of immunotherapy in the setting of dexamethasone administration also revealed significant worsening of clinical outcomes in patients treated with dexamethasone + immunotherapy, specifically PD-(L)1 therapy [92]. Moreover, another study showed that dexamethasone suppression can be overcome by strong CD28 stimulation or CTLA-4 blockade [93]. These are critical findings that must be considered going forward, because many GBM patients are on chronic or even permanent dexamethasone treatment to deal with the edema related to the tumor invasion and RT, and future clinical trials must deal with the physiologic effects of the steroid.

CTLA-4, also known as CD152, is a high-affinity receptor for B7 that induces negative costimulatory signaling on activated T cells [94]. It is upregulated on activated regulatory T cells (Tregs) and non-Treg T cells and supports an immunosuppressive environment by inducing immune tolerance [95]. CTLA-4 blockade has shown durable results in many types of cancer including melanoma, NSCLC, and renal cell carcinoma (RCC), possibly secondary to intratumoral Treg depletion [75, 96, 97]. The CTLA-4 blocker ipilimumab is currently being evaluated in GBM [NCT04323046, NCT04396860, NCT04817254]. Omuro et al. published the Phase I exploratory cohort of the CheckMate143 trial [NCT02017717], showing that ipilimumab was safe in combination with nivolumab [98]. Whether the CTLA-4 inhibitors will yield long-term advantage over the current standard of care remains to be seen. Further trials of various checkpoint inhibitors are ongoing and are listed in Table 1.

**Table 1:** Active trials involving immune checkpoint inhibitors

NCT	Phase	Intervention	Target population	Location	Sample size	Key outcomes
NCT02337686	Phase II	Pembrolizumab both pre- and postoperatively	Recurrent glioblastoma	USA	20	PFS, OS, ORR, toxicity, cytokine evaluation
NCT02617589	Phase III	Nivolumab/RT vs TMZ/RT	Newly diagnosed MGMT-unmethylated glioblastoma	USA	560	OS, PFS, tumor mutational burden comparison
NCT02667587	Phase III	Nivolumab/TMZ/RT vs placebo/TMZ/RT	Newly diagnosed MGMT-methylated glioblastoma	USA	716	PFS, OS
NCT02852655	Phase I	Neoadjuvant pembrolizumab vs placebo	Surgically accessible recurrent/progressive glioblastoma	USA	25	TIL density, adverse events, PFS
NCT02974621	Phase II	Cediranib/olaparib vs bevacizumab	Recurrent glioblastoma	USA	70	PFS, OS, adverse events, cytokine evaluation, tumor genetics
NCT03047473	Phase II	Adjuvant avelumab with TMZ/RT	Newly diagnosed glioblastoma	Canada	30	Adverse events, MRI evaluation, biomarker evaluation
NCT03158389	Phase I/II	Matches one of 7 drugs to patients (APG101, alectinib, idasanutlin, atezolizumab, vismodegib, Palbociclib, and temsirolimus) based on molecular markers after surgery, in addition to RT	MGMT-unmethylated glioblastoma	Germany	350	PFS, OS, adverse events
NCT03174197	Phase I/II	Atezolizumab/TMZ/RT	Newly diagnosed glioblastoma	USA	80	DLT, OS, adverse events, ORR, PFS
NCT03197506	Phase II	Neoadjuvant pembrolizumab and then adjuvant RT/TMZ/pembrolizumab	Newly diagnosed glioblastoma	USA	50	DLT, OS, adverse events, PFS, biomarkers
NCT03341806	Phase I	Avelumab (PD-L1 inhibitor) with or without MRI-guided laser interstitial thermal therapy	Recurrent glioblastoma	USA	13	DLT, ORR, PFS
NCT03426891	Phase I	Pembrolizumab/vorinostat (histone deacetylase inhibitor)/TMZ/RT in dose escalation	Newly diagnosed glioblastoma	USA	21	MTD, OS
NCT03493932	Phase I	Nivolumab with BMS-986016 (anti-Lag-3 antibody), with active monitoring by cerebral microdialysis	Recurrent glioblastoma	USA	20	IFN–γ levels, microdialysis safety, drug safety
NCT03532295	Phase II	Epacadostat (IDO-1 inhibitor) + INCMGA00012 (anti-PD1 antibody) given alone or in combination prior to RT/bevacizumab	Recurrent glioblastoma	USA	55	OS, PFS, neurological function, safety
NCT03718767	Phase II	Adjuvant nivolumab	IDH mutant glioma with or without hypermutator phenotype	USA	95	PFS, HRQOL, neoantigen burden
NCT03899857	Phase II	Study of standard of care with the addition of pembrolizumab for newly diagnosed GBM	Newly diagnosed glioblastoma	Switzerland	56	OS, PFS, treatment failure, PD-1 expression levels
NCT03925246	Phase II	Nivolumab in varying doses	Recurrent IDH mutant glioblastoma, prior TMZ/RT	France	43	PFS, OS, ORR, safety
NCT03961971	Phase I	MBG453 (anti-Tim3 antibody) and Spartalizumab (anti-PD1 antibody) given 1 week prior to SRS, then in an adjuvant fashion ongoing	Recurrent glioblastoma	USA	15	Adverse events, toxicity, PFS, OS, ORR
NCT04047706	Phase I	BMS 986,205 (IDO1 inhibitor) with nivolumab and TMZ/radiation	Newly diagnosed glioblastoma	USA	30	Adverse events, OS, PFS, MRI evaluation
NCT04145115	Phase II	Combination therapy with ipilimumab and nivolumab	Somatically hypermutated glioblastoma	USA	37	ORR, OS, PFS, adverse events
NCT04225039	Phase II	A Cohort = single priming dose of both drugs prior to RT, then adjuvant schedule for both drugs; B Cohort = neoadjuvant schedule of both drugs, then RT/resection, then adjuvant treatment	Recurrent glioblastoma	USA	32	ORR, adverse events, OS, PFS
NCT04323046	Phase I	Neoadjuvant Ipilimumab/nivolumab plus adjuvant nivolumab after surgery, vs placebo	Recurrent/progressive high-grade glioma in children/young adults	USA	45	Cell cycle gene changes, adverse events, OS, PFS
NCT04396860	Phase II/III	Phase II = ipilimumab alone; Phase III = ipilimumab and nivolumab; all patients received RT as well; active comparator is TMZ/TTF	Newly diagnosed MGMT-unmethylated glioblastoma, IDH wild type	USA	485	PFS, OS, adverse events, HRQOL, MGMT expression
NCT04608812	Phase I	OS2966 (mAb against CD28/b1 integrin) directly infused into the brain tumor and surrounding infiltrated brain	Newly diagnosed glioblastoma that has received resection and standard of care	USA	24	DLT, optimal dose, spatial distribution of drug, MRI evaluation
NCT04656535	Phase 0/I	AB154 (anti-TIGIT) and AB122 (anti-PD-1) in various combinations vs placebo control	Recurrent glioblastoma	USA	46	Adverse events, RNA sequencing, T cell changes
NCT04729959	Phase II	Tocilizumab ± atezolizumab, combination w/ surgery ± RT	Recurrent glioblastoma	USA	12	DLT, MTD, ORR, PFS, OS, adverse events
NCT04817254	Phase II	TMZ/RT and ipilimumab in two different dose regimens	Newly diagnosed glioblastoma or gliosarcoma	USA	48	OS, T cell responses, HRQOL
NCT04826393	Phase Ib	Dose escalation trial of ASP8374 (anti-TIGIT antibody) and cemiplimab (anti-PD1 antibody)	Recurrent high-grade glioma	USA	24	MTD, TIL, adverse events, PFS, OS
NCT04922723	Phase I/II	Adjuvant TMZ/RT plus daratumumab (anti-CD38)	Newly diagnosed glioblastoma	USA	16	DLT, OS, PFS, adverse events
NCT04952571	Phase II	Camrelizumab and bevacizumab	Recurrent glioblastoma	China	94	PFS, OS, ORR, control rate, functional outcomes, adverse events

While immune checkpoint inhibitors hold great promise for treating tumors in the primary and recurrent settings, their use is not without risk. Although widespread success has been reported when using ICIs against other cancers, many reports also describe therapy-induced immune-related adverse events (irAEs). This is a category of side effects encompassing a broad range of events from minor rashes to fatal pneumonitis and liver failure [99–101]. The National Comprehensive Cancer Network recently updated their guidelines on the diagnosis, monitoring, and management of adverse events related to checkpoint inhibitors [101], and it will be important for clinicians to monitor for irAEs, especially since treatment with TMZ has a significant risk of myelosuppression and many clinical trials offer standard of care with the addition of immune checkpoint blockade. These risks are reflected in the numbers of patients who suffer from irAEs in clinical trials. For example, an ongoing phase I/II clinical trial [NCT03174197] of the PD-L1 antagonist atezolizumab with TMZ and RT reported interim results showing that, while there was modest efficacy of the treatment, over half of the 60 patients enrolled thus far had Grade 3 or higher adverse events that were likely related to treatment [102]. These events were most commonly noted as elevated liver function tests and lymphopenia. An improved understanding of risks associated with ICIs and combination therapies will be critical to support treatment decision-making.

#### Innate immune cell-based therapies

The innate immune system is a key component to the immunosuppressive TME of GBM, helping to drive tumor immune escape and progression of the tumor. TAMs are pivotal cells in the TME. Microglia are the dominant brain parenchymal immune cells under healthy conditions and derive from embryologic progenitor cells that associate themselves with the CNS. Historically, it was believed that the immune cells in the cerebrospinal fluid and meninges were derived from circulating populations. However, according to recent work, both macrophages/monocytes and B cells in the meninges and cerebrospinal fluid can be traced back to local skull and vertebral bone marrow populations, which are distinct from the circulating pools of these cells [36, 103]. There is a significant difference between the healthy brain immune environment and the tumor-associated immune environment. In GBM, TAMs include some resident microglia, but overwhelmingly consist of invading bone marrow-derived macrophages [104–106]. However, resident microglia can take on a proinflammatory phenotype via a TGF-β dependent mechanism, and while they make up a small percentage of the TAMs, they provide an important stimulus to the tumor to allow progression [107]. The number and density of these cells within the GBM TME correlate with grade of glioma, with GBM having the highest proportion [108]. Previously, these TAMs were thought to express an M2 immunosuppressive phenotype because of their ability to secrete TGF-β and IL-10, but more recent studies have shown that they are a combination of M1 and M2 phenotypes, expressing both immunosuppressive (e.g., TGF-β, IL-10) and inflammatory (e.g., TNF-α, CXCL10) molecules, as well as factors that promote angiogenesis and breakdown of the extracellular matrix (e.g., VEGF, matrix-metalloproteinases) [104, 109–111]. TAMs also support the mesenchymal phenotype in GBM, through a variety of mechanisms. For instance, TAM’s secret oncostatin M (OSM) upregulates the OSM receptor on the tumor cells and activates STAT3 signaling [112]. This induces a mesenchymal transition of the GBM cells, increasing for tumor invasiveness. Animal models that knockout TAMs have reduced glioma invasion and tumor [105]. Thus, TAMs are critical for oncogenesis and a promising target for immunotherapeutic research.

Myeloid-derived suppressor cells (MDSC) also play a dynamic role in the immune response to GBM. MDSCs are a subset of myeloid cells (CD33^+^HLADR^−^) and are known to be increased in patients with cancer, including GBM [113, 114]. They exist in three subsets: neutrophilic (CD33^+^HLADR^−^CD15^+^), monocytic (CD33^+^HLADR^−^CD14^+^), and immature (CD33^+^HLADR^−^CD14^−^CD15^−^), each of which has a distinct infiltration pattern in the TME [114, 115]. MDSCs can induce T cell dysfunction via mechanisms including increased arginase production which leads to decreased L-arginine and reduced T cell function [116] and increased reactive oxygen species production [113]. MDSCs are increased in the blood of GBM patients, and their infiltration into the TME induces immunosuppressive effects [113–116].

One clinical study showed that neutrophilic MDSCs from the blood of newly diagnosed GBM patients had strong T cell suppressive effects in vitro, including reduced proliferation and reduced IFN-y production [115]. They also found that both blood-derived and tumor-derived MDSCs produce PD-L1 and can induce the expression of PD-1 from tumor-associated CD4 + effector memory T cells, functionally exhausting them and reducing their function [115]. Raychaudhuri et al. demonstrated that depletion of MDSCs from GBM patients partially restored the T cell proliferation and elevated IFN-γ in the blood [113]. Additionally, they showed that plasma levels of granulocyte colony stimulating factor (G-CSF) were increased in GBM patients, which is key for recruitment of the MDSCs to the tumor microenvironment. Another study demonstrated that macrophage migration inhibitory factor secreted by GSCs helps upregulate MDSC activity and promotes GBM immune evasion [117]. MDSCs can also affect B cell function. MDSCs also play a major role in B cell-associated immune suppression in the GBM microenvironment via transfer of membrane-bound PD-L1 to the B cells via both direct cell–cell contact mechanisms and through extracellular vessel signaling [118]. In this same study, 40% of the glioma samples were positive for regulatory B cells, which express PD-L1 and CD155 and produce immunosuppressive cytokines TGF-β and IL10 [118].

These preclinical studies informed a phase 0/I clinical trial using metronomic capecitabine as an immunomodulator in order to investigate the effects of chemotherapy on the TME following resection [NCT02669173]. Capecitabine has been shown to reduce intratumoral MDSCs when given in a low-dose, time-dependent fashion [117, 119]. Eleven patients received varying doses of capecitabine for 5–7 days prior to surgery for a recurrent GBM, followed by low-dose capecitabine and bevacizumab as maintenance therapy [119]. Preliminary reports found a decrease in the numbers of circulating MDSCs over time in the patients receiving higher doses as well as an increase in inflammatory infiltrate including CD8 + T cells and NK cells in the TME based on flow cytometry [119].

Dendritic cells (DC) may also serve as a therapeutic vehicle in GBM. DCs are antigen-presenting cells that phagocytose foreign (or self) antigens display them for detection by other immune cells, through a process called antigen cross-presentation [120]. They help generate immunity to foreign and auto-antigens and are vital in activating both the innate and adaptive immune responses in cancer [121, 122]. They activate the immune system by presenting antigens to T cells, directly interacting with B cells and NK cells, and by creating inflammatory and activating cytokines [122]. In cancer, they can acquire a tumor-infiltrating DC (TIDC) phenotype, which is broadly characterized as immunosuppressive with decreased antigen presentation and increased regulatory membrane ligands/receptor expression [122, 123]. They are present in the brain TME and are influenced by intratumoral expression of various factors, including fibrinogen-like protein-2 (FGL2) from the tumor cells and upregulation of Nrf2 in conventional DCs [124, 125]. Various preclinical models have demonstrated that DC activity can be upregulated by supplementing with stimulatory cytokines, such as IL12, double-stranded DNA segments, tetanus toxoid, and CCL3, which may portend a promising role for DCs in GBM therapy [126, 127]. Recently, DCs have become heavily developed as a cellular platform to deliver antigen-specific vaccines to GBM patients [121, 128].

Prior attempts at DC-directed therapy have yielded mixed results for GBM. Typically, producing a DC vaccine treatment involves incubating the autologous patient-derived DCs with the antigen(s) of interest in the presence of stimulating chemokines. These activated DCs are then reinfused back to the donor to induce their anti-disease effects. A variety of targets have been utilized, and many trials are ongoing. A common emerging target for DC therapy is the cytomegalovirus (CMV)-derived antigen pp65. CMV DNA has been demonstrated to be present in various cancers and has been implicated in oncogenesis and oncomodulation [129]. Pp65 and other CMV antigens have been proven to be expressed by about 90% of GBM samples, but not normal brain tissue [130, 131]. Thus, it is an attractive target for immunotherapies. Reap et al. treated CMV-seropositive patients with newly diagnosed GBM using a vaccine containing both CMV pp65-specific T cells and CMV pp65-RNA-loaded DCs and found that adding DCs to the regimen increased the T cell polyfunctionality compared to T cells only. With this polyfunctionality came improved survival, although the study was not powered to determine a difference between cohorts in OS or PFS [130].

A series of trials was performed for GBM with anti-CMV pp65 DC vaccines, each utilizing different pretreatment regimens before administration of the vaccine to patients. In the ATTAC-Td trial, Mitchell et al. treated newly diagnosed GBM patients with a DC vaccine against CMV pp65, after pretreatment with tetanus toxoid. These patients survived longer than expected, and three of them had not progressed at time of publication (over 36 months since their treatment) [127]. This led to a second trial from the same group, ATTAC-GM utilizing GM-CSF as the compared pretreatment, which was double the cohort size and showed a 30% long-term survival rate as well [132]. The addition of GM-CSF pretreatment to the vaccine showed significant benefit for the patients, thought to be secondary to immunological priming. Long-term analysis of these two trials showed that the median OS for ATTAC-Td and ATTAC-GM was 37.7 months and 38.3 months, which are significantly longer than historical cohorts and matched cohorts receiving standard of care [133]. Akasaki et al. treated newly diagnosed and recurrent GBM post-resection via cervical intradermal injection of fusion cells created from both autologous GBM cells and autologous DCs. PFS and OS in the recurrent group were 10.3 and 18.0 months, while newly diagnosed patients were 18.3 and 30.5 months, respectively [134]. The key takeaways from the trials listed thus far are that immunoreactive primers (GM-CSF, tetanus toxoid) are not only favorable but also required, because creating that inflammatory environment increases the likelihood of antigen presentation from the vaccine, activating the immune response.

Another popular DC vaccine strategy is the incubation of the patient-derived DCs with synthetic peptides based on analysis of tumor antigens and often includes several targets given the heterogeneous nature of GBM [135]. The DCs are created via isolation of monocytes from patient sera, then stimulation with IL-4, GM-CSF, then TNF-α, and finally by adding the peptides (derived from autologous tumor lysate or synthetically produced) to the DCs in a pulsed fashion to improve antigen presentation [136]. This technology has been standardized, and DC vaccines are now commercially available. For instance, ICT-107 is a six-peptide DC vaccine which has been developed for GBM patients [136]. It contains the peptides HER2, TRP-2, gp100, MAGE-1, IL13Rα2, and AIM-2, all of which have been shown to be overexpressed in GBM and by GSCs [136, 137]. A Phase I trial demonstrated clinical safety as well as improved median PFS and OS, 16.9 and 38.1 months, respectively [136]. Evidence of successful GSC targeting was noted via the decrease in CD133 positivity in patients with recurrence. A Phase II, placebo-controlled trial of the ICT-107 vaccine in 124 patients, however, only showed a 2.2 month increase in PFS of patients receiving the vaccine vs the placebo and did not show a significant difference in overall survival [135]. In this trial, patients received standard of care, then the vaccine weekly for 4 weeks, and then 12 months of adjuvant TMZ. Maintenance vaccinations occurred at 1, 3, and 6 months, then every 6 months thereon. Even though there was no OS improvement in the full cohort, there was suggestion that HLA-A1 + vs HLA-A2 + status of the patients along with MGMT promotor methylation status had significant effects on the outcome of the patients. This is likely related to difference in HLA recognition of the antigens. (gp100, HER2/neu, IL13Rα2, and TRP-2 are HLA-A2 + antigens, while MAGE-1 and AIM-2 are HLA-A1 + antigens.)

Although synthetic peptide DC vaccines have been given significant attention, trials of DCs primed with whole tumor lysate have been performed as well. Buchroithner et al. performed a Phase II study of Audencel, a whole tumor lysate DC vaccine against GBM, and found that there was no improvement in OS over the standard of care [138]. Liau et al. published a Phase III trial of DCVax-L, an autologous tumor lysate-pulsed DC vaccine, in 331 patients. Patients were randomized to either TMZ and placebo or TMZ and DCVax-L, with crossover to the vaccine cohort permitted upon recurrence. In the intention to treat analysis, mOS was 23.1 months postoperatively. In subgroup analyses, methylated MGMT (O6-methylguanine-DNA-methyltransferase) promotor patients had an mOS of 34.7 months, while MGMT-unmethylated patients had mOS of 19.8 months, and 186 patients had followed up > 36 months postoperatively. [139]. While these studies show mixed results, the capability of the vaccine to be customized to the patient’s own tumor cannot be understated. When this technology progresses, this may offer an avenue for personalized therapy.

DC vaccines remain promising because of their very low side effect profile as currently published and their high specificity to the antigens within the GBM. Their ability to be customized to the patient in a safe and efficient manner offers precision unparalleled by many other treatment types. Additionally, they can be combined with almost any other therapeutic drug to achieve many different goals. Their expansion into the sphere of oncology will be robust, and they will be offered for many different pathologies in the near future. The active trials for dendritic cell vaccines are listed in Table 2.

**Table 2:** Active trials involving myeloid cell-based therapies/vaccines

NCT	Phase	Intervention	Target population	Location	Sample size	Key outcomes
NCT00639639	Phase I	CMV pp65-LAMP mRNA-loaded DC vaccine with or without autologous lymphocyte transfer	Newly diagnosed glioblastoma, during recovery from TMZ-induced lymphopenia	USA	42	Feasibility and safety of DC vaccine, immune responses, DC tracking
NCT01204684	Phase II	Autologous tumor lysate-pulsed DC vaccine alone, with resiquimod, or with poly:ICLC	Newly diagnosed or recurrent high-grade glioma, prior resection at recruiting center	USA	60	Most effective combination of vaccine components, PFS, OS
NCT01567202	Phase II	RT/TMZ plus DC vaccine loaded with glioma stem cell antigens vs with placebo vaccine	Newly diagnosed glioblastoma, IDH1 wt, TERT mutated	China	100	ORR, OS, PFS
NCT02010606	Phase I	Antigens taken from GSC cells lines, pulsed with autologous patient DCs, in conjunction with TMZ/RT or optional bevacizumab depending on cohort	Newly diagnosed or recurrent glioblastoma	USA	39	Safety and tolerability, adverse events, PFS, OS, T cell activity
NCT02366728	Phase II	Adjuvant tetanus toxoid preconditioning followed by human CMV pp65-LAMP mRNA-pulsed autologous DC vaccine	Newly diagnosed glioblastoma, recently resected	USA	64	mOS, DC migration to lymph nodes, mOS/mPFS in CMV-seropositive vs seronegative patients
NCT02649582	Phase I/II	Autologous WT1 mRNA-loaded DC vaccine given after resection, with adjuvant TMZ	Newly diagnosed glioblastoma	Belgium	20	Safety and feasibility of vaccine production, adverse events, immunological response, clinical efficacy
NCT02820584	Phase I	Autologous tumor GSC lysate-loaded DCs alone	Newly diagnosed glioblastoma	Italy	20	Safety, vaccine production, OS
NCT03395587	Phase II	Autologous tumor lysate-loaded DC vaccine with adjuvant RT/TMZ vs standard of care	Newly diagnosed glioblastoma, IDH wt	Germany	136	OS, PFS, adverse events, functional outcome
NCT03548571	Phase II/III	Trivalent DC vaccine (survivin, hTERT, autologous tumor stem cell antigens) followed by TMZ, vs standard therapy alone	Newly diagnosed glioblastoma, IDH wt, MGMT unmethylated	Norway	60	OS, PFS, immunological response, adverse events
NCT03866109	Phase I/II	CD34-enriched hematopoietic stem cells and progenitor cells that express IFN-a2	Newly diagnosed glioblastoma, MGMT-unmethylated	USA	21	Tolerability and safety, hematologic recovery, max dose, PFS, OS, functional outcomes
NCT03879512	Phase I/II	Autologous tumor lysate-loaded DC vaccine, with prior cyclophosphamide (Treg depletion), with nivolumab/ipilimumab dual therapy followed by nivolumab monotherapy	Recurrent high-grade glioma in children and adolescents	Germany	25	OS, PFS, toxicity, Treg numbers, T cell responses, pathology correlation
NCT03927222	Phase II	CMV pp65-LAMP mRNA-loaded DCs with GM-CSF, TMZ, and Td toxoid	Newly diagnosed glioblastoma, MGMT unmethylated, CMV seropositive	USA	48	mOS, DC cell migration, cytokine expression, Treg increase, toxicity
NCT04201873	Phase I	Tumor lysate-loaded DC vaccine, pembrolizumab, poly-ICLC vs placebo/DC vaccine/poly-ICLC	Recurrent or progressive glioblastoma	USA	40	Cell cycle signature, expansion of TCR clones, adverse events, PFS, OS
NCT04277221	Phase III	Autologous DC vaccine loaded with tumor lysate with and without bevacizumab	Recurrent glioblastoma	Taiwan	118	OS, PFS
NCT04388033	Phase I/II	DC/glioma fusion vaccine plus IL-12 plus TMZ in the adjuvant phase	Newly diagnosed, recently resected glioblastoma	China	10	Adverse events, OS, PFS
NCT04552886	Phase I	TH-1 personalized DC vaccine with TMZ/RT after resection	Newly diagnosed glioblastoma	USA	24	Safety, toxicity, OS, PFS
NCT04801147	Phase I	Autologous tumor lysate-loaded DC vaccine after resection and TMZ/RT	Newly diagnosed glioblastoma	Italy	76	PFS, adverse events, immune response
NCT04888611	Phase II	Camrelizumab (anti-PD-1) with GSC antigen-loaded DC vaccine, vs placebo	Recurrent glioblastoma	China	40	OS, PFS, adverse events, exploratory biomarkers
NCT04963413	Phase I	Autologous DCs derived from PBMC loaded with RNA encoding the human CMV matrix protein pp65-flLAMP plus GM-CSF, with TMZ maintenance	Newly diagnosed glioblastoma, recently completed TMZ/RT	USA	10	Safety and feasibility of generating vaccine doses

#### Peptide vaccines

Another class of immunotherapies are peptide vaccines, which are created from published tumor-specific antigens and induce a cytotoxic T cell response against the tumor. For instance, epidermal growth factor receptor variant III (EGFRvIII) is tumor-specific deletion of a portion of the EGFR gene that causes constitutive activation of the receptor [140]. This mutation is present in nearly one-third of GBM, but not present in normal tissues, making it a true tumor-specific antigen and therapeutic target. In murine models, peptide vaccination against EGFRvIII results in a robust antitumor response and improved survival. The vaccine peptide rindopepimut has been synthetically created based on a small amino acid sequence surrounding the fusion site on EGFRvIII, conjugated to an immunogenic protein called keyhole limpet hemocyanin, that serves as an adjuvant and activates both the humoral and cellular immunity [141]. It was found to be potentially useful in GBM and has been used in several trials. For instance, the phase II ACT II trial showed promise, with no autoimmune reactions generated, and improved PFS and OS compared to historical controls [142]. A follow-up phase II trial, ACT III, found that anti-EGFRvIII antibodies were efficiently produced in patients, that PFS and OS were improved compared to historical cohorts, and that patients could safely receive rindopepimut treatment for an extended period of time, over 3.5 years in this study [143]. ACT IV, the randomized, double-blind phase III trial in newly diagnosed GBM, was unable to reproduce a statistically significant difference between rindopepimut vaccine-receiving patients and those receiving the placebo vaccine [144]. This may be related to selective pressure rindopepimut therapy placed on recurrent tumors to self-select for cells without an EGFRvIII mutation and immunologically escape the effects of the vaccine [142].

Rindopepimut was also trialed in the context of recurrent GBM. ReACT, a recently published phase II trial of bevacizumab plus rindopepimut or placebo in recurrent GBM, showed that patients receiving the peptide vaccine had longer OS, better overall response rate, and were able to come off of corticosteroid therapy more often compared to the placebo [140]. These improved responses correlated with higher anti-EGFRvIII titers in the trial. The effects of bevacizumab and the rindopepimut may synergize, secondary to bevacizumab’s known quality of improving immunologic responses in preclinical models [145, 146]. Rindopepimut continues to be studied, in concert with other immunotherapies, for GBM.

Other peptide vaccines are being investigated and show significant promise. Heat shock protein peptide complex-96 (HSPPC-96) is a common intracellular chaperone that binds tumor-associated antigens, and is able to channel antigens into the MHC class I cross-presentation pathway to prime antigen-specific CD8 + T cells [147–149]. Bloch et al. studied a vaccine against HSPPC-96 in a phase II study of 41 patients with recurrent GBM and found that it was safe (only one vaccine-related adverse event) and that there was 90% survival at 6 months and a median OS of 42.6 weeks [150]. Ji and colleagues trialed the HSPPC-96 vaccine combined with standard of care in a phase I study of 20 patients with newly diagnosed GBM. They reported no high-grade adverse events, with a median PFS of 11 months and median OS of 31.4 months [147]. It is clear that other potential targets exist within GBM, and these peptide or HSP-based vaccines can target them with the machinery of the patients’ native immune system.

DC technology offers the possibility of personalized peptide vaccines, made from autologous tumor cells that are lysed and used to generate a patient tumor-specific DC vaccine. In this method, the lysate content is analyzed in contrast to patient DNA and propensity to bind to various receptors on antigen-presenting cells. Kodysh et al. reported a phase I trial of a personalized peptide vaccine derived from autologous tumor lysate plus tumor-treating field therapy in 8 patients with recurrent GBM. Antigens were collected from tumor samples after initial biopsy or resection, and patients could receive up to 14 vaccines in total [151]. They reported that median overall survival has not been reached and that 12-month PFS and OS were 62.5% and 83.3%, respectively [151]. Another ongoing phase II trial that involves personalized tumor lysate vaccines was published by Bota et al., who utilized patient plus three standardized prior GBM patients’ antigens to create their vaccines, which were administered with GM-CSF, cyclophosphamide, and bevacizumab [152]. They demonstrated a median OS of 12 months for their vaccine plus bevacizumab group vs 7.5 months for placebo + bevacizumab [152]. A third group developed a personalized vaccine (GAPVAC) with both mutated and unmutated antigens based on tumor analysis and administered it to 16 patients, achieving a 29-month mOS and 14.2-month PFS [153].

Another group developed a personalized neoantigen vaccine based on whole-exome sequencing and administered it to eight patients in a prime-boost schedule. Six patients completed the prime-boost, and overall survival was 16.8 months in their cohort of newly diagnosed GBM [154]. There were few adverse events, and it was noted that when patients did not receive dexamethasone, there was significant CD4 + and CD8 + reactivity to the neoantigens, with a significant memory population, and a demonstrated ability to cross the blood–brain barrier based on T cell receptor analysis [154]. Dexamethasone-administered patients had a smaller degree of immunogenicity and worse response overall to the vaccine. This is an exciting finding but also potentially problematic, given the ubiquity of dexamethasone administration in GBM to treat cerebral edema. More work will need to be done to determine dexamethasone’s overall effects on vaccine response.

Peptide vaccines provide novel targets for immunotherapy and proof-of-concept studies to identify upregulated proteins in the tumor cells and targeting them with peptide vaccines. As the technology matures, there will be a wide range of options in place for patients and clinicians. Active clinical trials including peptide vaccines are included in Table 3.

**Table 3:** Active trials involving peptide and tumor lysate-derived vaccines

NCT	Phase	Intervention	Target population	Location	Sample size	Key outcomes
NCT01903330	Phase II	ERC1671 (tumor-derived cells and cell lysate) combined with GM-CSF and cyclophosphamide to stimulate the immune response, with bevacizumab, vs placebo injection with bevacizumab	Recurrent glioblastoma or gliosarcoma	USA	84	OS, PFS, immune response, adverse events
NCT02287428	Phase I	NeoVax (personalized neoantigen vaccine from autologous tumor lysate) with pembrolizumab with or without standard therapy	Newly diagnosed glioblastoma	USA	56	Safety, adverse events, number of actionable peptides, feasibility of vaccine administration
NCT03018288	Phase II	RT/TMZ plus pembrolizumab, with or without HSPPC-96 (heat shock protein peptide complex-96) peptide vaccine	Newly diagnosed glioblastoma	USA	90	OS
NCT03149003	Phase III	WT1 antigen-based peptide vaccine (DSP-7888) with concurrent bevacizumab vs bevacizumab alone	Recurrent or progressive glioblastoma with HLA-A2 positivity	Multiple countries	236	DLT, OS, PFS, adverse events
NCT03223103	Phase I	Personalized vaccine containing synthetic long peptides adjusted to the patient's tumor, with poly:ICLC and tumor-treating fields	Newly diagnosed glioblastoma immediately after TMZ/RT	USA	13	DLT, OS, PFS, ORR
NCT03382977	Phase I/IIa	Use of a pp65 peptide-containing vaccine, administered with GM-CSF for immune response improvement	Recurrent glioblastoma	USA	38	DLT, immunogenicity, anti-CMV immunity, Treg and myeloid changes, OS, PFS
NCT03650257	Phase II	gp96 vaccine received after TMZ/RT	Newly diagnosed high-grade glioma	China	150	OS, PFS, T cell changes
NCT03665545	Phase I/II	IMA950, a standardized peptide vaccine, plus poly-ICLC for immunogenicity	Recurrent glioblastoma	Switzerland	24	Adverse events, PFS, OS, TIL density, T cell responses
NCT04013672	Phase II	Pembrolizumab, with SurVaxM, sargramostim, and montanide ISA 51 in combination	Recurrent glioblastoma	USA	40	PFS, safety, and tolerability
NCT04116658	Phase Ib/IIa	EO2401 (therapeutic peptide vaccine based on tumor-associated antigens and microbiome-derived peptides) with and without nivolumab and bevacizumab	Recurrent or progressive glioblastoma with HLA-A2 positivity	Multiple countries	52	Safety and tolerability, OS, immunogenicity
NCT04280848	Phase I/II	Peptide vaccine with telomerase-derived antigens, with Montanide ISA 51 (vaccine adjuvant)	Recurrent or progressive, MGMT-unmethylated glioblastoma	France	28	Immunogenicity via T cell responses
NCT04642937	Phase I	Combination therapy with anti-CD200AR ligand, imiquimod, and GBM6-AD tumor lysate vaccine	Recurrent glioblastoma	USA	24	MTD, adverse events, OS, PFS
NCT04808245	Phase I	Peptide vaccine against H3K27M antigens, along with atezolizumab and imiquimod	Newly diagnosed H3K27M-mutated gliomas	Germany	15	Toxicity, immunogenicity, PFS, OS
NCT04842513	Phase I	Vaccine (XS15) containing specific immunomodulatory proteins after standard of care	Newly diagnosed IDH wt, HLA-A2 positive, MGMT-methylated glioblastoma after resection	Germany	15	Adverse events, immunogenicity changes

#### Lymphocyte-based therapy

In addition to the innate immune system, tumor effects on the adaptive immune system play an important role in gliomagenesis. Most important, and most heavily investigated, is the role of tumor-derived T cell alterations in progression and immune evasion. Tumor cells produce an environment in which immune escape functions are much easier to achieve, through aberrant Treg activation in the TME, as well as generalized decrease in cytotoxic T cell effector functions [155]. Additionally, GBM and other gliomas can sequester peripheral circulating T cells in the bone marrow, causing relative lymphopenia compared to non-GBM controls [156]. Since GBM has a small number of neoantigens/low mutational burden [23, 26], it can reverse-engineer the natural mechanisms of self-tolerance that are imprinted into healthy T cells [155]. GBM and other cancers can induce apoptosis in invading CD4^+^ and CD8^+^ T cells through Fas/FasL signaling [155, 157, 158]. Not surprisingly, a high proportion of CD8^+^ T cells in the TME confers a large survival benefit over lower levels of infiltrate [159]. However, one group found that CD8^+^ T cells, while initially rapidly expanded in a trial of a dendritic cell vaccine, did not have a durable response in patients who received TMZ chemotherapy [160]. This suggests that the CD8^+^ T cell environment in the TME is fluid and subject to many different forces. For example, recently, Mathewson et al. published an analysis of 31 patients with IDH mutant GBM examining potential new inhibitory targets expressed on tumor-infiltrating T cells. They found that KLRB1 (an NK cell gene that encodes CD161) has an inhibitory effect when expressed by these T cells and that genetic silencing of this and other NK cell genes can lead to improved antitumor response [161]. Glioma cells can also select for CD8 + T cell-resistant clones within their tumor mass and can undergo immunoediting processes and acquire myeloid transcriptional programs to avoid attack from CD8 + T cells and further avoid immune attack [162, 163]. As time progresses, more abilities of glioma to avoid adaptive immunity will be unearthed.

Tregs influence tumor immune escape by creating an immunosuppressive environment via the production of TGF-β and IL10, which decrease the ability of CD8 + T cells to effect their responses against cancer cells [164]. The GBM cells promote the survival and activity of Tregs within the TME via expression of CCL2 (a Treg cytokine) [104, 165], LSP-1 [166], STAT3 [167–169], HIF-f1α (hypoxia-inducible factor 1α) [170], and IDO (indolamine-2,3,-dioxygenase) [171, 172]. These changes make it very difficult for the body to mount an effective immune response to the GBM, because of significant immunosuppression and inability to activate cytotoxic T cell responses to the aberrant tumor cells. Considering these many functions, Tregs are promising potential targets for GBM immunotherapy.

Chimeric antigen receptor T cells (CAR-T) are genetically engineered T cells that possess artificial receptors which are targeted to an antigen of choice [173, 174]. These cells are able to bind tumor-specific antigens without a reliance on natural mechanisms of antigen presentation, allowing for fully primed CAR-T cells to infiltrate tumors and perform effector functions. First-generation CARs were designed only to induce cytolysis, but not proliferation and sustained activation of T cells [175]. More recent developments have created CARs that induce T cell activation, proliferation, and cytokine release. Depending on the costimulatory pathway through which these CARs activate the T cells, they are deemed second generation (with a 4-1BB or CD28 co-stimulation domain) or third generation (containing both 4-1BB and CD28 co-stimulation domains) [173, 175]. CAR-T cells have yielded responses in B cell malignancies including acute lymphocytic leukemia (ALL) and diffuse large B cell lymphoma (DLBCL) and have received FDA approval in these cancer types [176, 177]. This has expanded their study, now being used on a variety of antigens across a high range of solid malignancies, including GBM.

Various preclinical studies have determined that CAR-T cells can be an effective therapeutic adjunct for GBM. Clinical trials are ongoing. The most studied targets for CAR-T in GBM so far have been EGFRvIII, HER2, and IL-13αR2, and these have published clinical trial results [178–182]. Weiss et al. demonstrated in an immunocompetent mouse model that NKG2D-directed CAR-T cells infused peripherally can navigate to the site of the tumor in the brain, produce little systemic side effects, and can cure a fraction of the mice in vivo. In vitro, they observed high IFN-γ production and increased cytolytic activity of these CAR-T cells against glioma cell lines [183]. They also observed a synergistic effect with RT in these mice. Yang et al. also used NKG2D-directed CAR-T cells to target GBM, showing high elimination of GBM cells and the glioma stem cells within the tumor [184]. The preclinical investigation has been extensive, and this has led to significant clinical trial efforts as well. A summary of active clinical trials for CAR-T and other lymphoid cell therapy in GBM is presented in Table 4.

**Table 4:** Active trials involving T cell-based therapies

NCT	Phase	Intervention	Target population	Location	Sample size	Key outcomes
NCT02661282	Phase I/II	Autologous, CMV antigen-specific T cells with TMZ	Newly diagnosed or recurrent glioblastoma	USA	27	MTD, immunological effects, PFS, OS
NCT03170141	Phase I	Intravenous administration of immunogene-engineered autologous T cells after depletion with fludarabine/cyclophosphamide	Recurrent glioblastoma, prior treatment with standard of care	China	20	Safety, response rate, OS, PFS
NCT03344250	Phase I	Adjuvant use of bi-armed activated T cells against EGFR and CD3, with TMZ/RT	Newly diagnosed glioblastoma	USA	18	MTD, immune changes, OS, PFS, pathology correlation
NCT03347097	Phase I	Engineered autologous T cells expressing a full anti-PD1 antibody, infused after TMZ/RT	Newly diagnosed glioblastoma	China	40	Adverse events, PFS, OR
NCT03389230	Phase I	Memory-enriched CAR-T therapy specific to HER-2 with a truncated CD19	Recurrent high-grade glioma	USA	42	Adverse events, DLT, CAR-T detection, PFS, OS
NCT03726515	Phase I	EGFR-vIII directed CAR-T cells combined with pembrolizumab	Newly diagnosed MGMT-unmethylated glioblastoma, EGFRvIII +	USA	7	Adverse events, OS, PFS, ORR
NCT04003649	Phase I	Nivolumab, ipilimumab, and IL13Rα2-directed CAR-T therapy together after resection of recurrence	Recurrent glioblastoma	USA	60	Adverse events, DLT, feasibility of therapy, OS
NCT04045847	Phase I	Intratumoral CD147 CAR-T cells	Recurrent glioblastoma, prior treatment with standard of care	China	31	Adverse events, DLT, MTD, clinical activity, CAR-T detection
NCT04077866	Phase I/II	TMZ alone vs TMZ with B7-H3 CAR-T cells via intraventricular/intratumoral injection	Recurrent or progressive glioblastoma with B7-H3 positivity	China	40	OS, adverse events, PFS, MTD, CAR-T detection, pharmacokinetics
NCT04165941	Phase I	Genetically modified, TMZ-resistant γδ T cells intratumorally following the completion of TMZ/RT	Newly diagnosed glioblastoma	USA	12	MTD, PFS, OS, biological activity
NCT04214392	Phase I	Chlorotoxin-directed CAR-T cells given via dual delivery	Recurrent glioblastoma with MMP2 positivity	USA	36	DLT, CAR-T cell detection, cytokine levels, OS, PFS, antigen expression levels
NCT04717999	Phase I	Intraventricular NKG2D CAR-T cell therapy	Recurrent Glioblastoma	Taiwan	20	DLT, ORR, PFS

In a phase I trial of 10 patients with recurrent EGFRvIII + GBM, O’Rourke et al. demonstrated that EGFRvIII-directed CAR-T cells were safe and effective. After a single peripheral infusion, they were able to show that the CAR-T cells had engrafted in the peripheral circulation, trafficked to the brain (confirmed by surgical samples post-infusion), and were able to proliferate (confirmed via Ki-67 testing) [185]. However, they determined that the GBM responded by downregulating EGFRvIII expression, avoiding therapeutic pressure. To overcome this, one group infused IL-12 directly into the tumor in a preclinical model, to encourage TME changes as well as increased EGFR-vIII-CAR-T efficacy [186]. With a similar purpose, Choi et al. and Nakazawa et al. used CRISPR/Cas9 to genetically remove PD-1/PD-L1 sensitivity in their EGFR-vIII-CAR-T cells, leading to highly increased responses in their murine model without affecting the individual effector functions of the CAR-T cells [187, 188]. Brown et al. utilized CAR-T cells directed against IL-13Rα2 in a patient with metastatic, recurrent GBM. They directly infused the CAR-T cells into the intraventricular space in the patient’s CNS. The response was profound (total regression of all lesions) and durable (7.5 months after initiation of therapy) and was coupled with a low side effect profile. Levels of several inflammatory cytokines also increased tenfold over baseline levels [179]. These CAR-T cells are being used in an ongoing clinical trial [NCT02208362]. Another group directed CAR-T at chlorotoxin, a scorpion-derived peptide that is nontoxic to humans, that has been shown to bind with high selectivity to GBM and not to surrounding normal brain [189]. These CAR-T cells showed in a preclinical model highly precise targeting to the GBM and regression of tumor without significant side effects in orthotopic xenograft models [189]. Tang et al. demonstrated that CAR-T directed at B7-H3, a transmembrane protein that is highly expressed on DCs and TAMs in GBM (among other cancer types), was able to provide significant tumor reduction in a preclinical model as well [190]. There are many promising candidates for CAR-T therapy targets in GBM, but large-scale clinical trials showing sustainable results are yet to be performed.

As with any other medical advancement, side effects can temper progress; this has been true of CAR-T therapy as well. There has been significant use of CAR-T in severe refractory hematologic malignancies, with reports of significant side effects, most commonly cytokine release syndrome (CRS) and neurologic toxicities [191, 192]. CRS after CAR-T is associated with fever that can progress to arrhythmias, hypotension, hypoxia and even respiratory failure, as well as other minor toxicities [191]. Neurologic toxicity, also called CAR-T-related encephalopathy syndrome (CRES), can manifest as a variety of neurologic or behavioral changes and can be as severe as cerebral edema and death [191].

Natural killer (NK) cells are innate lymphocytes that play a role in tumor surveillance and elimination via direct cytotoxic effects and through production of cytokines to inhibit cancer metastasis and proliferation [193]. The consensus is that NK cells have significant activity against GSCs [194–196] and that they reduce systemic metastasis propensity of the GBM cells, meaning that they are potential therapeutic vehicles for GBM. Lee et al. demonstrated that NK cells derived from human donors were able to prevent and/or eliminate systemic metastatic deposits of GBM in an orthotopic murine model [197]. A paper from Friebel et al. showed that both cytotoxic and immature NK cells infiltrate GBM, with a smaller proportion of each type correlating to reduced grade of tumor [198].

Recently, NK cells have been deployed in preclinical models of cancer in a therapeutic role, similar to CAR-T cells, because of their innate ability to identify/attack abnormal self-cells and ignore healthy cells, through MHC-I recognition [193]. Additionally, they can be created with a chimeric antigen receptor (CAR-NK cells) and infused like T cells, allowing for similar types of therapies. It has been speculated that NK cells would be a strong candidate for immunotherapeutics because of the high expression of NKG2D ligands on both the GBM cells and the glioma stem cells that promote tumorigenesis [199]. Close et al. demonstrated that NK cells preferentially attack glioma stem cells rather than neural progenitor cells in an in vitro assay when stimulated by IL-15. Additionally, this group determined that tumor-infiltrating NK cells had reduced expression of NGK2D, NKp30, CD2, and other NK cell-specific surface receptors known to participate in activation [200]. Crane et al. determined that GBM can also induce NKG2DL expression in TAMs and peripheral blood monocytes, leading to overall down-regulation of the NKG2D receptor and reduced NK cell effector functionality [201]. These findings suggest that GBM is able to robustly down-regulate the effector mechanisms of NK cells that infiltrate the TME. For example, Shaim et al. published that TGF-β signaling through the αv integrin axis causes down-regulation of NK cell function against glioma stem cells, but this can be rescued through treatment aimed at this signaling interaction [196].

However, in light of GBM’s ability to weaken their immune signaling, NK cells have also been implicated as a key player in the potentiation of various immunotherapies in GBM. As an example, Pellegatta et al. published an analysis of their phase II DC cell vaccine trial that demonstrated highly expanded NK cell infiltrates in patients that had PFS > 12 months [160]. As NK cell technology continues to expand, we will likely see combinations of personalized NK cells and other cells, including CAR-T cells, DC cells, or others, to treat cancer with the full force of the immune system.

#### Viral vector therapy

Viral therapy represents another route of intensive research in regard to immune therapy for GBM, and many clinical trials have been performed so far for different mechanisms of vector action. Oncolytic viral vectors take advantage of a virus’ native ability to replicate and lyse cells and combines it with genetically engineered safeguards to ensure that tumor cells are lysed and invoke a strong immune response in the region of the cancer, via release of neoantigens and damage-associated molecular patterns [202–205]. Moreover, the virus can be created with the goal of delivering genetic therapy, key inflammatory mediators, or other molecules that will aid the patient’s native immune system in reacting to the tumor [206]. Often, virus selectivity for cancer cells relies on proteins that are overexpressed by the cancer but are poorly expressed or unexpressed in the normal surrounding cells, but can take the form of other targeting mechanisms as well. Most commonly, the viruses are herpesviruses, reoviruses, pox virus, or adenoviruses which are subjected to varying degrees of genetic engineering [203, 207]. The research continues to expand the available vectors, however, with two recent publications highlighting the use of the Zika virus for targeting of GBM tissue [208, 209]. The evidence for oncolytic viral therapy in GBM has been increasing in depth over the last two decades, with fifteen active clinical trials at the time of this review. Excellent reviews of completed trials have been published [203, 207], and this review will focus on highlights of major trials and active trials. Active trials are listed in Table 5.

**Table 5:** Active trials involving oncolytic virus-based therapies

NCT	Phase	Intervention	Target population	Location	Sample size	Key outcomes
NCT00634231	Phase I	AdV-tk (oncolytic adenovirus) intratumorally plus valacyclovir immediately after resection	High-grade glioma or recurrent ependymoma in children	USA	8	Safety, adverse events, OS, PFS, immune response
NCT02062827	Phase I	M032 (oncolytic HSV-1) delivered intratumorally via MRI-guided infusion	Recurrent high-grade glioma	USA	24	MTD, PFS, OS
NCT02457845	Phase I	G207 (oncolytic HSV-1) with RT intratumorally	Progressive high-grade brain tumors in children	USA	12	Safety, adverse events, immunologic response, virologic shedding, PFS, OS
NCT02798406	Phase II	DNX-2401 (oncolytic adenovirus) delivered intratumorally; with combination pembrolizumab	Recurrent glioblastoma or gliosarcoma	USA	49	ORR, OS, duration of response
NCT03072134	Phase I	NSC-CRAd-Survivin-pk7 (neural stem cells loaded with oncolytic adenovirus) delivered intratumorally after resection	Newly diagnosed high-grade glioma	USA	13	MTD, MRI evaluation of tumor response
NCT03152318	Phase I	rQNestin34.5v.2 (oncolytic HSV-1) given intratumorally after biopsy; cyclophosphamide given preop	Recurrent high-grade glioma	USA	56	MTD, MRI changes, viral shedding, viremia, HSV1 antibody response
NCT03576612	Phase I	AdV-tk (oncolytic adenovirus) intratumorally plus nivolumab plus oral valacyclovir and TMZ/RT	Newly diagnosed high-grade glioma	USA	36	Adverse events, OS, PFS, immune profiling, cytokine profiles
NCT03636477	Phase I	Intratumoral Ad-RTS-hIL-12 (adenovirus encoding human IL-12) after resection, veledimex (IL-12 immunotherapeutic activator), and nivolumab	Recurrent glioblastoma	USA	21	Safety, tolerability, ORR, PFS, OS, pharmacokinetics
NCT03657576	Phase I	Dose escalation and safety trial of genetically engineered HSV C134	Recurrent high-grade glioma	USA	24	Adverse events, PFS, OS, HSV titer, WBC composition
NCT03714334	Phase I	Intratumoral injection of DNX-2440 (oncolytic adenovirus)	Recurrent glioblastoma or gliosarcoma	Spain	24	Adverse events, OS, ORR
NCT03911388	Phase I	G207 (oncolytic HSV-1) delivered intratumorally, postoperative RT	Recurrent posterior fossa brain tumors in children	USA	15	Safety, tolerability, immunologic response, viral shedding, PFS, OS
NCT04006119	Phase II	Ad-RTS-hIL-12 (adenovirus encoding human IL-12), veledimex (IL-12 immunotherapeutic activator), and cemiplimab (anti-PD-1)	Recurrent glioblastoma	USA	40	Safety and tolerability, OS, PFS, ORR, immune responses
NCT04406272	Phase II	VB-111 (oncolytic adenovirus) intravenously preop and intraop, vs placebo groups, with bevacizumab	Recurrent glioblastoma	USA	45	TIL density, DLT, PFS, OS
NCT04479241	Phase II	Intratumoral PVSRIPO (poliovirus) followed by IV pembrolizumab	Recurrent glioblastoma	USA	30	ORR, duration of response, adverse events, OS, PFS, biomarker evaluation
NCT04482933	Phase II	Intratumoral G207 (HSV-1) with postoperative RT	Recurrent high-grade glioma in children	USA	30	OS, adverse events, virologic shedding, immune responses

With regard to recent publications of completed trial data, a few recent ones in particular are key to the understanding of the current state of viral therapy for GBM. Desjardins et al. published their phase I trial of 61 patients with recurrent GBM who received intratumoral infusions of PVSRIPO, a recombinant poliovirus that recognizes CD155, the poliovirus receptor which is highly expressed on GBM cells [210, 211]. They demonstrated that 21% of patients had an overall survival that was greater than 36 months in their trial, significantly higher than historical controls [210]. These data informed the creation of a phase II clinical trial of PVSRIPO with the addition of anti-PD1 therapy with pembrolizumab for patients with recurrent GBM [NCT04479241]. Another key trial published by Lang and colleagues utilized DNX-2401, an oncolytic adenovirus with tumor selectivity via inactivation of the E1A gene which prevents viral replication in normal cells which have a functional Rb (retinoblastoma) signaling pathway [202, 212]. This trial involved 37 patients with recurrent malignant glioma, and in 25 patients in the safety and dose escalation arm 20% of patients survived > 3 years after the administration of the viral vector [202]. They found the treatment to be safe (no dose-limiting toxicity was found) and were able to demonstrate that there was a shift to a T_H_1 response, overall reduction in CD4^+^ T cells, and an overall increase in CD8^+^ T cells, indicative of an improved immune response [202].

Geletneky et al. published another phase I trial of rat H-1 parvovirus (H-1PV) used intratumorally in 18 patients with recurrent GBM and found that they were able to achieve an overall survival of 72% at 1 year, and a median overall survival of about 15 months [213, 214]. They had no dose-limiting toxicities and found a mildly increased T cell response in the further resected tumors in this cohort [213, 214]. In a trial of another species of virus, Markert et al. evaluated the effects of G207, an oncolytic HSV-1 (herpes simplex virus-1) virus that has key deletions not allowing the viral ribonucleotide reductase to function outside tumor cells, in nine patients with recurrent, progressive GBM. They demonstrated that there were no dose-limiting toxicities and patients survived a median 7.5 months from their inoculation with the virus [215]. G207 has also been studied in pediatric high-grade gliomas, as recently published by Friedman and colleagues as a phase I clinical trial. They treated 12 patients, with or without radiation, via intratumoral infusion with G207, and found clinical responses in 11 of 12 patients, with median overall survival of 12.2 months and 4 of 11 patients still alive at 18-month follow-up [216, 217]. The data from this trial informed a larger phase II clinical trial currently ongoing [NCT04482933].

The group of Fares and colleagues set out to do things a little differently. They recently published a phase I, first-in-human, dose escalation trial of a new oncolytic adenovirus NSC-CRAd-S-pk7, which is delivered to the tumor via a neural stem cell vehicle [218]. This mechanism takes advantage of the natural affinity of neural stem cells to cross the blood brain barrier, enter the tumor bed, and proliferate there [219, 220]. Fares et al. treated their patients with the neural stem cells loaded with viral vector, and then the patients received standard of care 10–14 days later. Their median PFS was 9.1 months and median OS was 18.4 months, with no treatment-related deaths [218]. The results of this trial will inform large phase II and III clinical trials, per the report of the authors.

Adenovirus vectors can also be used to deliver immunotherapy to brain tumors. For example, Kieran et al. performed a phase I clinical trial of gene-mediated cytotoxic immunotherapy (GMCI) using the adenovirus AdV-tk, which was injected into the tumor bed at the time of surgery, and followed it with 14 days of valacyclovir [221]. The purpose of the valacyclovir is to induce cell death via direct targeting of the infected tumor cells, and immunogenically prime the TME. Their results are promising. Of eight total patients receiving the GMCI, three patients lived longer than 24 months after injection, with the two longest-lived surviving 37.3 and 47.7 months [221]. This approach combines the actual cytotoxic effects of the viral vector with an immune system-stimulating effort, with the combination theoretically improving the global response to the tumor.

Viral vectors are an ongoing area of research in the sphere of GBM. They hold great promise as potential cures, with several examples of long-standing response and high degrees of quality of life in these patients, and have little in the way of side effects thanks to their exceptional specificity to the tumor cells. However, there remains the task of identifying which patients will respond to viral vector therapy up front, to allow for the patients to be funneled to other treatment types if they are able. Some research has been performed regarding this specific topic and is summarized in an excellent review [222], but the biomarkers and other factors that may predict response to oncolytic viral therapy are yet to be reliably elucidated.

#### Nucleic acid-based therapy

Lastly, therapies that fall under the umbrella of genetic material-driven mechanisms of action are burgeoning and have some exciting clinical trials worth noting in this review. For example, oligonucleotide-based therapy offers a wide range of therapeutic options for cancer. Because of the ability to engineer the oligonucleotide exactly to a genetic sequence that would be unique to the tumor rather than the native cells, there can be exact specificity for this type of agent in a treatment course. One such agent is IMV-001, which is an siRNA antisense oligonucleotide that targets the insulin-like growth factor-1 (IGF-1) receptor, a constitutively overexpressed oncogenic receptor in GBM that offers resistance to apoptosis and radiation for tumor cells [223, 224]. This drug was combined with autologous GBM cells in a phase I clinical trial of 33 patients with newly diagnosed GBM [225]. The analysis showed a median PFS of 11.6 months overall and 17.1 months in patients receiving the highest dose. In patients who were eligible for the Stupp protocol, a median overall survival of 38.2 months was noted [225].

Another example is the phase I trial utilizing two engineered DNA plasmids (INO-5401 and INO-9012) in combination with an anti-PD-1 antibody cemiplimab to deliver therapy [NCT03491683]. INO-5401 is a plasmid encoding hTERT, WT-1, and PSMA, while INO-9012 encodes IL-12, and they are given via electroporation to ensure their uptake [226] hTERT refers to human telomerase reverse transcriptase gene which is commonly mutated and overactive in GBM [227]. WT-1 is Wilms’ tumor-1, an oncoprotein that is highly expressed in GBM and is considered a tumor-associated antigen [228, 229]. PSMA refers to prostate-specific membrane antigen, which is found in the neovasculature within the body of a GBM [230]. The grouping, together with cemiplimab, is theorized to activate the immune system’s response against these specific antigens, which are not normally expressed on healthy cells. This combination was given to 52 patients, and in cohort A (adjuvant RT/TMZ only, no maintenance), there was 84% survival at 12 months, and T cell evaluation demonstrated responses to the plasmids [226]. Cohort B’s results (cohort A treatment (chemoradiation) plus maintenance chemotherapy with TMZ have yet to be published.

Nucleic acid-based therapies hold great promise because they offer the ability for highly specific targeting of tumor genes and gene products. The recent success with mRNA vaccine for COVID-19 underscores the potential for mRNA technology in cancer immunotherapy [231]. There is a lot more to be learned about these therapies and their potential efficacy and/or side effects in vivo in humans. A large number of clinical trials will likely open in the next decade as an increasing number of biotechnology companies gain and perfect the ability to manufacture these compounds. Current active trials are listed in Table 6.

**Table 6:** Active clinical trials involving nucleic acid-based therapies

NCT	Phase	Intervention	Target population	Location	Sample size	Key outcomes
NCT02766699	Phase I	Nanoparticles containing doxorubicin tied to bispecific antibodies (EGFR(V)-EDV-Dox) that are specific to the tumor cells of the patients	Recurrent glioblastoma	USA	20	Safety, MRI evaluation, recommended phase 2 dose, OS
NCT03491683	Phase I/II	INO-9012 (DNA plasmid for IL-12), INO-5401 (WT1 antigen, PSMA, hTERT genes) delivered via electroporation of cells, in combination with cemiplimab, with RT/TMZ	Newly diagnosed glioblastoma	USA	52	Adverse events, OS, change in T cell phenotypes
NCT03750071	Phase I/II	VXM01 (DNA plasmid vaccine for VEGFR-2) and avelumab (anti-PD-L1)	Recurrent glioblastoma	Germany	30	Adverse events, PFS, OS, MRI response, duration of response
NCT04485949	Phase IIb	IGV-001 (IGF-1R antisense oligonucleotide with autologous cell antigens) vs placebo with TMZ/RT	Newly diagnosed glioblastoma	USA	93	PFS, OS, MGMT status effect, functional changes, adverse events
NCT04573140	Phase I/II	Autologous tumor mRNA and pp65 LAMP mRNA-loaded liposomal vaccine	Newly diagnosed pediatric high-grade glioma and adult glioblastoma	USA	28	Production feasibility, safety, MTD

### Future perspective

#### Future of Immunotherapy

Although a wide range of immunotherapeutic interventions have shown promising results in preclinical models, many of these have not translated into effective or durable responses in the clinic. Many interventions focusing on combination immunotherapy can be used to increase the immune response; these strategies in GBM have been reviewed elsewhere [232]. The principle and practice of combination immunotherapy in general oncology has also been comprehensively reviewed recently [205]. Below, we highlight some key examples of preclinical data and active clinical trials in the field of GBM and immunotherapy.

Hung and colleagues used murine GBM models to demonstrate blocking both PD-1 and T cell immunoreceptor with Ig and ITIM domains (TIGIT), which is upregulated specifically in the GBM and cervical lymph nodes of both humans and mice, and demonstrated increased survival over PD-1 blockade alone [233]. They found that combining multiple routes of immune stimulation overcame some of the mutational ability of GBM. Another group demonstrated that targeting CXCR4 and PD-1 concurrently showed promise in murine models, with the combination regimen leading to decreased MDSC infiltration and improved CD4/CD8 T cell ratios in the tumor [234].

There have been extensive studies combining immune checkpoint blockade with immune-activating viral therapy as well. Speranza et al. combined anti-PD-1 treatment in a murine glioma model with intratumoral infusion of an adenovirus vector designed to lyse tumor cells and increase immune response [235]. This strategy demonstrated higher survival rate and T cell memory responses upon tumor re-challenge [235]. Saha et al. combined anti-PD-1 and anti-CTLA-4 antibodies were used with an IL-12 expressing oncolytic herpes simplex virus to cure GBM in a GSC-based murine model [236].

Several clinical trials examining combination immunotherapy are underway. In the TEM-GBM trial (NCT03866109), hematopoietic stem cells are transduced with a lentivirus that drives IFN-α expression in Tie-2-positive monocytes [237]. This combines a novel cell-based therapy and viral delivery of genetic therapy, in addition to immunomodulatory cytokine therapy. This trial has not yet reported any dose-limiting toxicities [237]. Another ongoing trial [NCT04003649] combines anti-IL13Rα2 along with nivolumab and ipilimumab in patients with recurrent GBM. This strategy combines CAR-T cells, as previously shown effective by Brown and colleagues [178, 238], with the promising effects of ICIs. This will theoretically prime the TME and down-regulate tumor-associated lymphocytes while simultaneously allowing for CAR-T mediated cytotoxicity against the tumor. Both trials are excellent examples of how the science will progress from our present state.

In the future, autologous glioma cell lysate and other personalized medicine tactics will be used to attack the tumor at an individual, tumor-specific level. Many personalized peptide and dendritic cell-based therapies are being evaluated in clinical trials [including NCT04888611, NCT04201873, NCT03223103, NCT01204684, NCT02820584, NCT03395587, NCT03879512, NCT04277221, NCT04801147]. As this technology develops, there will be a commensurate increase in the ability of modern oncology to prolong the survival of glioma patients. Additionally, personalized medicine capabilities are unleashed with the advancement of oligonucleotide and other nucleic acid-based therapies, including the mRNA vaccine technology popularized with the coronavirus vaccines of the current pandemic.

Due to the rise of immunotherapy and many novel agents, clinicians must take great care to appraise all the available clinical trials and weigh their potential benefits against traditional chemotherapy and radiation. This involves identifying patients that are interested and eligible for clinical trial participation. Key factors affecting participation in clinical trials include presence of American Society of Clinical Oncology (ASCO)-related comorbidities (e.g., hypertension, Alzheimer’s disease, and prior cancer), travel distance required to receive treatment, amount of paperwork and number of visits related to the trial, and fear of randomization, among many others [239–241]. The responsibility for enrolling patients who are interested in clinical trials falls primarily on the clinician. Having a knowledge of the various options for clinical trials, their potential side effects, and the centers offering them allows a clinician to ensure that the patient can be well informed and make a choice on their care for themselves. As the understanding of the immune system progresses, new and innovative potential therapies will present themselves to oncology. Clinicians must stay abreast of the literature and understand the options for their patients, to provide the best possible care for their present and future patients.

## Conclusion

The rapid adoption of immunotherapy in the sphere of oncology shows the ability of the translational medicine apparatus to take basic science advancements and implement them safely and effectively in humans. Clinicians must use these advancements to provide continually improved care to their patients, and as the range of options increases, it becomes more difficult to stay ahead of the myriad clinical trials and newly approved drugs each year. As the populace becomes more informed and more highly educated, a wider breadth of background knowledge will allow the physician or provider to correctly guide the patient toward treatments that will improve their survival, hopefully with good quality of life. We hope that this review provides the opportunity for providers to familiarize themselves with the current state of the science on immunotherapy of GBM.

## Data Availability

Not applicable.

## Abbreviations

ALL: Acute lymphocytic leukemia
ASCO: American Society of Clinical Oncology
ATRX: Alpha thalassemia/mental retardation syndrome X-linked
BBB: Blood–brain barrier
CAR: Chimeric antigen receptor
CMV: Cytomegalovirus
CNS: Central nervous system
CRES: CAR-T-related encephalopathy syndrome
CRS: Cytokine release syndrome
CT: Computed tomography
CTLA-4: Cytotoxic T-lymphocyte antigen-4
DC: Dendritic cell
DLBCL: Diffuse large B cell lymphoma
EGFR: Epidermal growth factor receptor
EGFRvIII: Epidermal growth factor receptor variant III
FGFR: Fibroblast growth factor receptor
FDA: Food and Drug Administration
FLAIR: Fluid attenuated inversion recovery
HIF-f1α: Hypoxia-inducible factor 1α
HSPPC-96: Heat shock protein peptide complex-96
HSV: Herpes simplex virus
ICI: Immune checkpoint inhibitor
IDH: Isocitrate dehydrogenase
IDO: Indolamine-2,3-dioxygenase
IFN-α: Interferon-α
IGF-1: Insulin-like growth factor-1
IL: Interleukin
irAEs: Immune-related adverse events
G-CSF: Granulocyte colony-stimulating factor
GARP: Glycoprotein-A repetitions predominant
GBM: Glioblastoma
GMCI: Gene-mediated cytotoxic immunotherapy
GM-CSF: Granulocyte–macrophage colony-stimulating factor
GSC: Glioma stem cell
KPS: Karnofsky performance scale
LAP: Latency-associated peptide
MDSC: Myeloid-derived suppressor cell
MGMT: O6-methylguanine DNA methyltransferase
MRI: Magnetic resonance imaging
mOS: Median overall survival
NF1: Neurofibromatosis type 1
NK: Natural killer
NSCLC: Non-small cell lung carcinoma
ORR: Objective response rate
OS: Overall survival
OSM: Oncostatin M
PD-1: Programmed death-1
PD-L1: Programmed death-ligand 1
PDGFRA: Platelet-derived growth factor receptor α
PFS: Progression-free survival
PSMA: Prostate-specific membrane antigen
PTEN: Phosphatase and tensin homolog
RCC: Renal cell carcinoma
Rb: Retinoblastoma
RT: Radiation therapy
TAM: Tumor-associated macrophage
TCR: T cell receptor
TERT: Telomerase reverse transcriptase
TGF-β: Transforming growth factor-β
TIDC: Tumor-infiltrating dendritic cell
TIGIT: T cell immunoreceptor with Ig and ITIM domains
TIL: Tumor-infiltrating lymphocyte
TLR3: Toll-like receptor 3
TME: Tumor microenvironment
TMZ: Temozolomide
TNF-α: Tumor necrosis factor alpha
Treg: T regulatory cell
VEGF: Vascular endothelial growth factor
WHO: World Health Organization
WT-1: Wilms’ tumor 1
